# Diagnostic Value of Real-Time Elastography in Diagnosing Lymph Node Metastasis of Skin Cancer

**DOI:** 10.7759/cureus.10997

**Published:** 2020-10-17

**Authors:** Suzan Onol, Ozay Ozkaya

**Affiliations:** 1 Radiology, University of Health Sciences Turkey, Prof. Dr. Cemil Taşçıoğlu City Hospital, Istanbul, TUR; 2 Plastic and Reconstructive Surgery, Ozay Ozkaya Clinic, Istanbul, TUR

**Keywords:** real- time elastography, lymph node, squamous cells carcinoma, malignant melanoma

## Abstract

Objective

The purpose of this study was to examine the diagnostic performance of real-time tissue elastography in detecting lymph node involvement in skin cancers.

Methodology

We retrospectively analyzed B-mode sonography and real-time elastography (RTE) images of 70 lymph nodes from 34 patients diagnosed with squamous cell carcinoma (SCC) or malignant melanoma (MM). In the B-mode examination, the appearance or loss of the hilar architecture in the lymph node, contour lobulation, and the presence of focal cortical thickening were evaluated. Elastography scores were classified according to the ratio of soft and hard areas across the lymph node on a 4-point scale system. Largely soft lymph nodes were scored as “1” and largely hard lymph nodes were scored as “4”.

Results

When patients with SCC and MM were evaluated together, the sensitivity of elastography was 94%, specificity was 70%, and the accuracy rate was 86% in detecting lymph node involvement. When both tumor groups were evaluated separately, for SCC, the sensitivity of elastography was 90%, specificity was 61%, and the accuracy rate was 80% in detecting lymph node involvement. When the receiver operating characteristic (ROC) curve was taken, the area under the curve (AUC) was 0.78 for SCC. Elastography showed full compliance with pathology in lymph node metastases of MM and the AUC was 1.00.

Conclusions

Based on our findings, RTE provides important contributions to B-mode ultrasonography (USG) in evaluating lymph node metastases of skin cancers.

## Introduction

Skin cancer is the most common cancer type among all cancers [1]. According to the National Comprehensive Cancer Network (NCCN) data, it is predicted that one in every five people in the world will have skin cancer at some point in their lives. Among skin cancers, squamous cell carcinoma (SCC) accounts for 25% of the diagnosis [2] and malignant melanoma (MM) makes up 2% [3]. The most important factors determining the surgical and oncologic treatment in both SCC and MM are tumor size (T), lymph node involvement (N), and the status of systemic metastases (M). Accurate evaluation of regional lymph nodes preoperatively is crucial for not only the decision-making process regarding sentinel lymph node biopsy (SLNB) or complete lymph node dissection but also for determining as to which patients should receive adjuvant therapy. However, difficulties are often encountered in the evaluation of superficial lymph node involvement before surgery, especially in the presence of non-palpable lymph nodes [4]. Ultrasonography (USG) is a fast, sensitive, easily accessible, and cost-effective imaging method and plays an important role in superficial lymph node imaging. Real-time elastography (RTE) is a newly developed modern USG method. Based on the elasticity and stiffness of the tissues, it plays a role in the differentiation of malignant and benign lesions [5,6]. With the technological developments in recent years, elastography can reveal focal changes in normal-sized lymph nodes.

The elastographic examination has been previously used in the differential diagnosis of breast, thyroid, prostate, liver, and cervix lesions. Elastography has also been investigated in many studies in the evaluation of superficial lymph nodes [7-9]. In this study, we aimed to analyze the efficacy of RTE in malignant and benign lymph node differentiation in patients with skin cancer.

## Materials and methods

Patient selection

Seventy lymph nodes from 34 patients (21 males, 13 females; mean age: 59.5 years; age range 31-88 years) who were diagnosed with skin cancer by biopsy between January 2012 and July 2017 were referred to the ultrasonography department of the Radiology Clinic of Prof. Dr. Cemil Taşçıoğlu City Hospital from the plastic surgery clinic of the same hospital for regional lymph node evaluation. Apart from the demographic characteristics of the patients, data on skin cancer type, B-mode USG results, RTE results, and lymph node pathology results were analyzed retrospectively.

Final diagnosis

The reference standard for the final diagnosis of reactive or metastatic lymph nodes was histopathologic findings. Pathology results of SLNB or neck dissection of patients' lymph nodes were compared with B-mode USG patterns and elastography scores. For this retrospective study, approval was obtained from the Ethics Committee of Istanbul Prof. Dr. Cemil Taşçıoğlu City Hospital.

USG technique

One radiologist who had 12 years of experience with conventional B-mode sonography and two years of experience in elastography performed the examinations. The patients' B-mode and RTE examinations for lymph nodes were performed in the same session, in supine patient positions, using a 5-13-MHz linear probe (HI VISION Avius®; Hitachi Medical Corporation, Tokyo, Japan). 

The B-mode sonographic examination was based on the criteria of size, shape, border, echogenicity, and hilum status. Lymph node size (short-axis diameter), shape (short/long axis ratio), cortex thickness, contour lobulation, presence and location of hilus, and echo pattern were routinely recorded with B-mode examinations. The elastographic records of sonographically normal lymph nodes and lymph nodes with focal thick cortex, with asymmetric or lacking hilus, and those having focal contour lobulation were examined.

Elastographic examinations were performed by applying manual rhythmic compressions on the lymph node upon the vertical direction using the same device and probe. Elastograms were examined on the screen in dual-mode, synchronously with real-time B-mode images. Surrounding fatty tissue with the lymph nodes was taken into the region of interest (ROI). The amplitude and frequency of the rhythmic compressions were monitored on the scale on the device screen, and a compression pattern of uniform intensity was followed. Real-time tissue elasticity was recorded with a color chart. Soft regions were coded with red, regions with average stiffness with green, and hard regions with blue. An average of five cine-loop images was taken for the examined lymph nodes.

Interpretation of images

On B-mode sonography, lymph nodes with a short axis greater than 10 mm were considered suspicious and included in the study. Increased short-to-long-axis ratio and ballooning of a lymph node were also accepted as probable malignant criteria. Irregular border, heterogeneous echogenicity, and absence of hilum were noted for malignant infiltration. Round-shaped lymph nodes with asymmetric hilus or without hilus, those having central hilus but presenting focal cortical thickening and irregularity, and those showing challenging shape were included in the study. Massive lymphadenopathies that protruded out of the screening area of the probe and prominent cystic necrotic lymphadenopathies that were certain to be malignant in the B-mode USG examination were excluded.

Lymph node elastographic analysis was performed on a simplified 4-point scale based on the study by Choi et al. [10]. The distribution ratio of hard areas (blue areas) and soft areas (green area) in lymph nodes was examined. Accordingly, scores 1 and 2 were accepted as soft (most likely benign), and scores 3 and 4 were regarded as hard (most likely malignant) (Table 1).

**Table 1:** Elastography scoring system for lymph nodes

Elastography score	Degree of hardness	Elastography view
1	Soft	Mostly green areas and less than 10% blue areas
2	Mostly soft	Mostly green areas and less than 45% blue areas
3	Mostly hard	Mostly blue areas and less than 45% green areas
4	Hard	Mostly blue areas and less than 10% green areas

Lymph nodes that were suspicious for malignancy in B-mode imaging or with scores of 2, 3, and 4 in the elastographic examination were marked on the skin before surgery or biopsy.

Statistical analysis

Data were presented as frequency and percentages. Nominal variables were analyzed using the Chi-Square test with Yates’s Continuity Correction and Fisher's exact test. The compatibility between pathology and elastography was evaluated using the Kappa test. Diagnostic test results [sensitivity, specificity, positive predictive value (PPV), negative predictive value (NPV), accuracy] and 95% confidence intervals were recorded. The limit of significance was taken as a p-value of <0.05 and it was considered as bidirectional. The analyses were performed using the NCSS 10 software program, 2015 (NCSS, LLC, Kaysville, UT). The receiver operating characteristic (ROC) curves and area under the curve (AUC) for elastography in the differentiation of reactive and metastatic lymph nodes were calculated in SCC and MM.

## Results

In our study, 70 lymph nodes of 34 patients were examined; 26 lymph nodes were taken from inguinal, five from the axillar, and 39 from the cervical region. The primary skin tumor was MM in 13 patients (21 of 70 lymph nodes) and SCC in 21 patients (49 of 70 lymph nodes). The primary tumor locations were head and neck region in 21 patients, extremities in 11 patients, back in one patient, and breast skin in one patient.

Pathologically, 24 of 70 lymph nodes were identified as benign and 46 as malignant. The median elastography score was 3 for the metastatic group and 2 for the non-metastatic group. The comparison of elastography scores and histopathologic results is shown below (Table 2).

**Table 2:** Comparison of elastography scores and histopathologic results

Elastography score	Metastatic	Non-metastatic
	n=46	n=24
1	1 (2.17%)	4 (16.67%)
2	1 (2.17%)	13 (54.17%)
3	15 (32.61%)	6 (25%)
4	29 (63.04%)	1 (4.17%)

**Figure 1:**
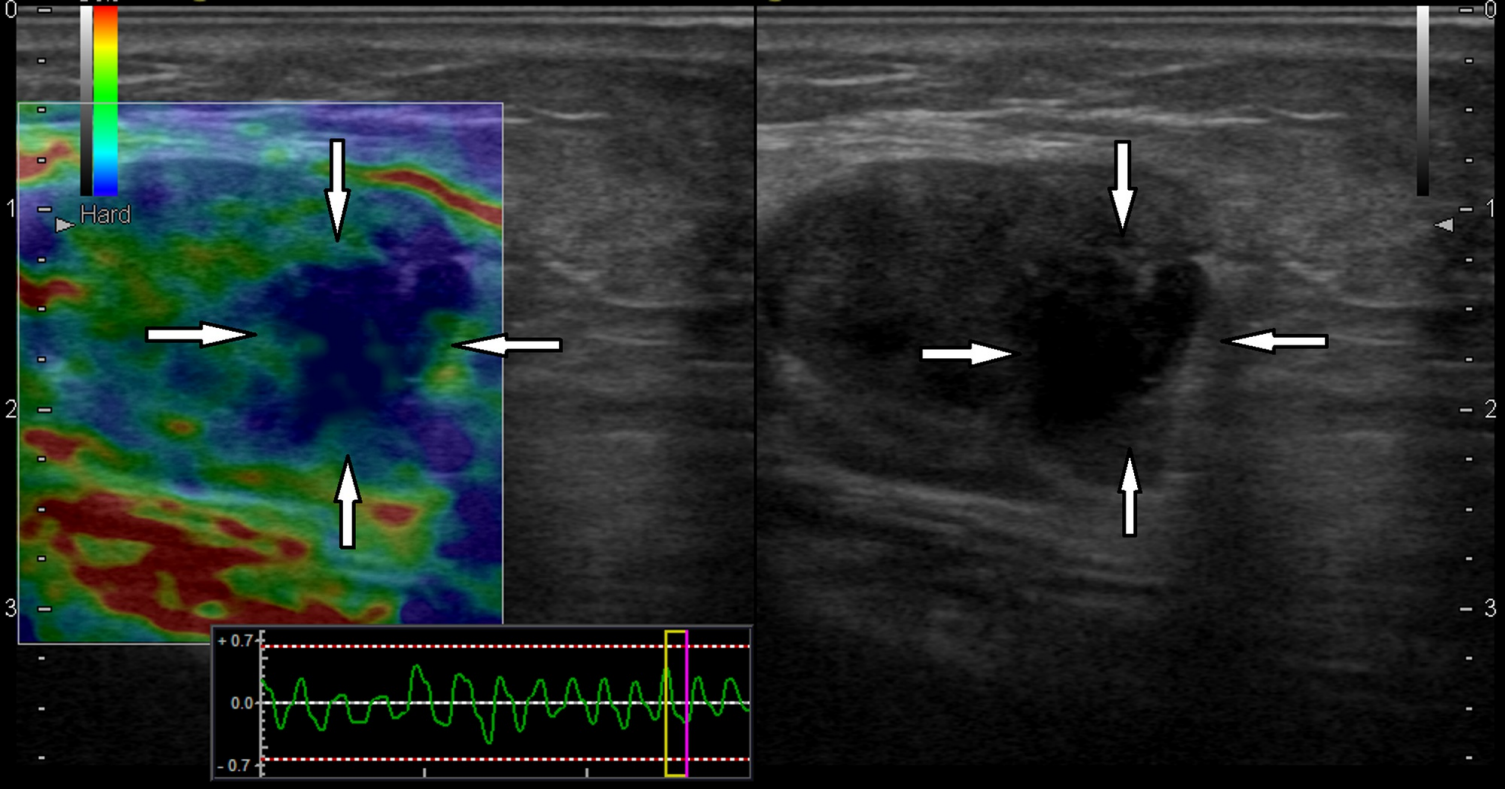
A 77-year-old male with a diagnosis of malignant melanoma of the left ankle In the left inguinal lymph node longitudinal sonogram, the lymph node had the heterogeneous round hypoechoic appearance and lobulated contour with irregularity in the B-mode examination (white arrows on right). In the elastography examination (left), there were metastatic hard areas with extension beyond the contour (white arrows on left). Histopathologically, the lymph node was diagnosed as metastatic with pericapsular spread

Histopathologically, in the MM group, five of the 21 lymph nodes were detected as reactive and 16 as melanoma metastases. The elastography score of six of the metastatic nodes was 3, and that of 10 of them was 4 (Figure 1).

Table 3 lays out histopathologic and elastographic correlations in the MM group.

**Table 3:** Lymph node evaluation in the malignant melanoma group

Elastography score	Lymph node pathology in malignant melanoma (n=21)	
	Reactive (n=5)	Metastatic (n=16)
1	-	-
2	5	-
3	-	6
4	-	10

**Figure 2:**
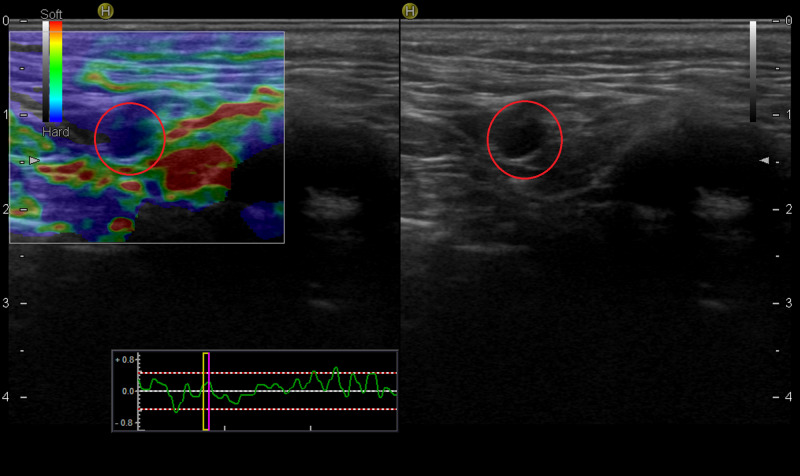
A patient with a lesion in the scalp diagnosed as squamous cell carcinoma The lymph node, which was marked before surgery, on the left side of the neck at level 2, was hypoechoic and round in the B-mode examination (red circle on right), with a score of 4 and hard in elastography (red circle on left). The pathologic diagnosis was granulomatous lymphadenitis

Histopathologically, in the SCC group, 17 of 49 lymph nodes were diagnosed as reactive and 30 as metastatic. The elastography score of 19 out of 30 metastatic lymph nodes was 4, that of nine out of 30 was 3, one out of 30 was 2, and the elastography score of one was 1. Two lymph nodes with scores 3 and 4 were diagnosed as granulomatous lymphadenitis (Figure 2).

**Figure 3:**
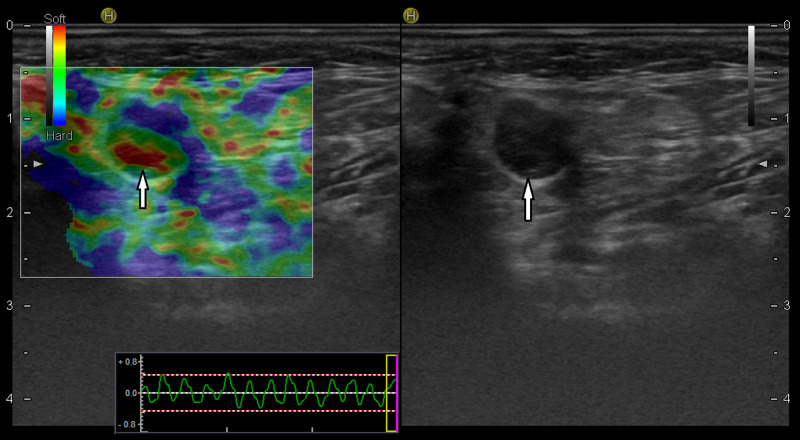
A 72-year-old woman with squamous cell carcinoma on the left upper lip skin The lymph node on the left side of the neck at level 2 contained round, hypoechoic weak microcystic openings in the B-mode examination (white arrow on right). In elastography (left), the lymph node score was 2, and the central section with cystic areas was markedly soft (white arrow on left). Histopathologically, metastatic involvement was detected in the lymph node

One metastatic lymph node with an elastography score of 2 contained microcystic changes (Figure 3).

Table 4 shows histopathologic and elastographic correlations in the SCC group.

**Table 4:** Lymph node evaluation in the squamous cell carcinoma group

Elastography score	Lymph node pathology in squamous cell carcinoma (n=49)		
	Reactive (n=17)	Metastatic (n=30)	Granulomatous lymphadenitis (n=2)
1	4	1	-
2	8	1	-
3	5	9	1
4	-	19	1

In both tumor groups, no statistically significant correlation was found between elastography and contour lobulation, and focal cortical thickening and loss of hilar architecture in the lymph nodes (p=0.099 and p=0.053, respectively). Table 5 shows the comparison of histopathologic results between the elastographically benign and malignant groups.

**Table 5:** Comparison of histopathologic results between elastographically benign (score 1-2) and malignant (score 3-4) lymph nodes

Histopathology	Elastography-benign (score 1-2)	Elastography-malignant (score 3-4)
Metastatic (n=46)	2	44
Non-metastatic (n=24)	17	7

There was a significant difference between metastatic and non-metastatic lymph node elastography scores (p<0.001). The median score in the metastatic group was 4 (29 lymph nodes, 63.04%). The median score in the non-metastatic group was 2 (13 lymph nodes, 54.17%). The sensitivity of RTE in metastatic lymph node involvement in both tumor groups was 94%, specificity was 70%, and accuracy was 86%.

**Figure 4:**
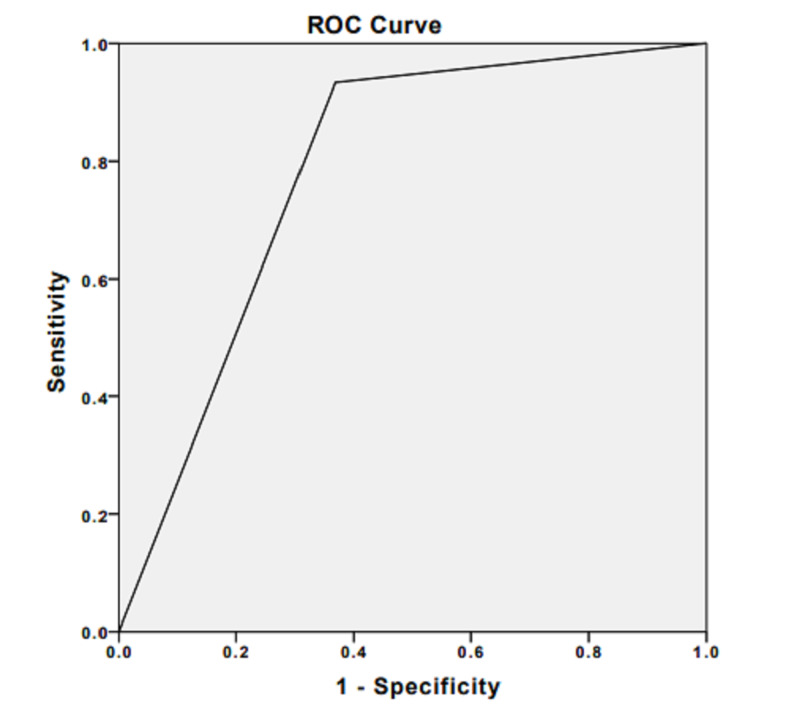
Receiver operating characteristic curve for elastography in malignant lymph nodes of squamous cell carcinoma Area under the curve is 0.78

When evaluated separately for SCC and MM, RTE was found to be fully compatible with pathology in MM lymph node metastases (K: 1 p<0.001). The AUC for elastography was 1.00. A statistically significant concordance between pathology and RTE was also found in SCC lymph node metastases (p<0.001). RTE sensitivity for SCC was 90%, specificity was 61%, and the accuracy rate was 80%. The ROC curve for elastography in SCC is shown in Figure 4.

## Discussion

RTE is a relatively new USG method that measures the elasticity of tissues with manual pressures applied through the USG probe. Elasticity is the response of the tissues, which varies from tissue to tissue to mechanical pressure. In addition, it is known that elasticity varies in different conditions such as inflammation, fibrosis, necrosis, and malignancy in the same tissue [8,10]. The stiffness of malignant tissues increases rather than normal tissues. In elastography, it is aimed to perform malignant/benign differentiation by measuring the response of tissues to compression performed with a USG probe [11].

Previously, studies on breast masses [12] and thyroid gland pathologies [13] have been conducted. Furthermore, there are many studies in the literature on the evaluation of the metastatic lymph nodes using RTE [14-17]. However, elastography studies of skin cancers, especially cutaneous SCC, are still scarce.

Detecting the presence of lymph node metastasis in skin cancers is mandatory in the decision-making process for surgical dissection, and it actually determines the prognosis. In cutaneous SCCs, the five-year survival is as high as 92% in early-stage limited nodal disease. In the presence of lymph node metastasis, the five-year survival decreases to 30% [4]. In recent years, radiologic evaluation of lymph node involvement has been recommended in patients with clinically high-risk SCC, even if there is no palpable lymph node [18].

Regional lymph node evaluation is also very important in patients with cutaneous MM before and after surgery [19,20]. In progressive MM, it is foreseen that lymphatic metastasis will develop within three years of the first diagnosis in two-thirds of patients. In the presence of a single metastatic lymph node, the five-year survival has been reported to decrease to 53% [21].

Cutaneous SCC develops as a result of the malignant proliferation of keratinized cells of the epidermis. The sensitivity of the elastography increases as the metastatic lymph nodes show significant stiffness because of intense keratinization. In a study by Alam et al., approximately 67% of neck lymph nodes were detected as SCC metastases. The sensitivity of RTE was reported as 83%, specificity as 100%, and accuracy rate as 89% [8]. Our results were similar to the results of Alam et al. However, in a similar study, Lo et al. showed that 77% of neck lymph nodes were detected as SCC metastases with a sensitivity of 63-67% and specificity of 56-57%, which were slightly lower compared to our findings [9]. More recently, in the study by Tanaka et al. on SCC, the sensitivity, specificity, and accuracy of RTE were found to be 100% in the detection of metastatic lymph nodes [22]. These different results in the literature may be attributed to the different parameters selected in the examinations in different studies. Currently, there is no broad consensus on elastography scoring. Some authors use a 4-grade scoring system while others use a 5-grade scoring scale. In addition, the lymph node locations in different regions in these studies may have affected the results. We think that an elastography scoring system consisting of a simplified 4-point scale, such as the one we used in this study, greatly facilitates the evaluation method.

Regarding lymph node metastases of MM, Ogata et al. found the sensitivity to be 92%, specificity to be 100%, and accuracy to be 95% for elastography score 4 in their study with 20 lymph nodes from 12 patients [23]. Hinz et al. showed that the sensitivity was 90% and the specificity was 76% in their study performed with 42 lymph nodes [24]. Tanaka et al. reported the sensitivity of RTE as 43%, specificity as 100%, and accuracy as 82% for 23 MM metastases [22]. Our results for MM metastasis are higher than those observed in the literature. The majority of MM lymph nodes being in the inguinal region may have influenced our results.

Metastatic involvement in the lymph node occurs through afferent lymphatics through cellular infiltration that begins at the periphery of the lymph node before the lymph node grows. At this stage, additional information is needed to evaluate changes observed with B-mode USG. We think that RTE reliably gives this additional information, especially in MM metastases. In addition, in our study, subcapsular metastatic deposits were detected in two patients as marked hard areas coded with intense blue color. These foci were also detected as prominent hypoechoic areas that created focal cortical thickening in B-mode images. The central echogenic hilar architecture in these lymph nodes was encoded in green in the RTE examination. Similarly, focal deposits were identified in two patients in the MM series of Hinz et al. [24]. In the case report of Aoyagi et al., focal cortical metastatic focus in the inguinal lymph node was detected by RTE in an 80-year-old female patient who was diagnosed as having invasive SCC in the right lower extremity [25]. We think that RTE is very important in detecting focal subcapsular deposits and contributes to B-mode examinations. Moreover, we found no statistically significant relationship between elastography and contour lobulation and loss of hilar architecture. This finding reveals that RTE and B-mode USG images must be interpreted together.

In our study, seven lymph nodes were evaluated as false-positive in terms of malignancy with elastography. Two of these lymph nodes were pathologically granulomatous lymphadenitis. Similarly, in the studies done by He et al. [26] and Rozman et al. [27], patients with mediastinal tuberculous lymphadenitis were evaluated as false-positive in terms of malignancy due to having hard lymph nodes in endobronchial sonoelastography examinations. It was thought that this might be a result of tissue hardening in tuberculous lymphadenitis due to calcium deposits and intense fibrosis. Currently, RTE for tuberculous lymphadenitis does not give clear results and additional studies are needed, especially from endemic regions.

In our study, two lymph nodes were evaluated as false-negative. One of these lymph nodes was a cutaneous SCC metastasis and included microcystic openings in the pathologic examination. Goddi et al. defined microcystic openings in normal prostate tissue with intermediate elasticity in their study [28]. We think that the microcystic openings caused the malignant lymph node to be identified as false-negative in our study.

In the literature, it was also noted in the study by Ginat et al. [29] that lesions with focal necrotic areas might be confused with cysts in breast lesions. In the study by Ishibashi et al. [30], it was revealed that necrosis-containing lymph nodes could give false-negative results. In our study, we think that we excluded lymph nodes with significant necrosis in B-mode imaging, and prevented more possible false-negative results.

Our study has some limitations. Primarily, it was retrospective in nature. The number of patients was low due to inaccessible patient records and files that did not comply with the study technique. Different anatomic locations (cervical, axillary, inguinal) of the lymph nodes led to a heterogeneous group. Another limitation was that the elasticity ratio (strain ratio) calculations between normal and malignant lymph nodes were not performed in patients and there was no quantitative evaluation related to strain ratios. This was largely due to the retrospective nature of the study. RTE is an operator-dependent examination, and interobserver compatibility should be considered as an important parameter in future prospective studies.

## Conclusions

Based on our findings, we conclude that RTE provides important contributions to B-mode USG in evaluating lymph node metastases of skin cancers as an inexpensive, easily accessible imaging method. The method can directly guide surgeons in the decision-making process related to SLNB or lymph node dissection in the presence of small lymph nodes, even in cases of non-palpable ones in patients with high-risk cutaneous SCC. In patients with MM, RTE is thought to play a complementary role to B-mode imaging, especially in the detection of subcapsular focal metastatic involvement.
